# Catecholamine Derivatives as Novel Crosslinkers for the Synthesis of Versatile Biopolymers

**DOI:** 10.3390/jfb14090449

**Published:** 2023-09-01

**Authors:** Manickam Sugumaran, Jason J. Evans

**Affiliations:** 1 Department of Biology, University of Massachusetts Boston, Boston, MA 02125, USA; manickam.sugumaran@umb.edu; 2 Department of Chemistry, University of Massachusetts Boston, Boston, MA 02125, USA

**Keywords:** dopa, dopamine, N-acyldopamines, tunichromes, dopyl proteins, sclerotization, melanization, quinones, quinone methides, catecholamines, antioxidants, antibiotics, elastic polymers, bioinspired polymers

## Abstract

Catecholamine metabolites are not only involved in primary metabolism, but also in secondary metabolism, serving a diverse array of physiologically and biochemically important functions. Melanin, which originates from dopa and dopamine, found in the hair, eye, and skin of all animals, is an important biopolymeric pigment. It provides protection against damaging solar radiation to animals. N-Acetyldopamine and N-β-alanyldopamine play a crucial role in the hardening of the exoskeletons of all insects. In addition, insects and other arthropods utilize the melanogenic process as a key component of their defense systems. Many marine organisms utilize dopyl peptides and proteins as bonding materials to adhere to various substrata. Moreover, the complex dopa derivatives that are precursors to the formation of the exoskeletons of numerous marine organisms also exhibit antibiotic properties. The biochemistry and mechanistic transformations of different catecholamine derivatives to produce various biomaterials with antioxidant, antibiotic, crosslinking, and gluing capabilities are highlighted. These reactivities are exhibited through the transient and highly reactive quinones, quinone methides, and quinone methide imine amide intermediates, as well as chelation to metal ions. A careful consideration of the reactivities summarized in this review will inspire numerous strategies for synthesizing novel biomaterials for future medical and industrial use.

## 1. Introduction

Catecholamines are an important group of compounds generated from the amino acid tyrosine. They are key components of both primary metabolism and secondary metabolism. The primary metabolites, dopa, dopamine, norepinephrine, and epinephrine, are extremely crucial for all living organisms. Several of these compounds and their derivatives are used widely as drugs for the treatment of various ailments. Tyrosine and dopa are also converted into a vast array of secondary metabolites that are very important for various organisms. Lignin and tannins produced by plants are essential for the survival of all plant species. Marine organisms and arthropods synthesize numerous catecholic compounds. The catecholic (*o*-diphenolic) group attributes special reactivities to catecholamines due to the ease with which they undergo oxidation to highly reactive quinonoid products. Studies carried out extensively over the past fifty years have brought to light another important aspect of catecholamines, *viz*., the reactivities through their side chains. Armed with these two reactivities, catecholamines form a variety of biomaterials that possess amazing properties. The importance of such molecules has only been recognized recently, although their involvement in biomaterial formation was well established years ago. Several simple derivatives possess antioxidant as well as antibiotic properties. Some form novel crosslinking agents for the construction of tough exoskeletons of insects and other arthropods. Some possess a combination of both antibiotic and crosslinking capabilities. Some are used as novel gluing materials to bond to solid substrata. In this review, advances made in the past fifty years on the reactivities of various catecholamines with respect to biomaterial formation are discussed in detail. Examination of the reactivities of catecholamine derivatives, especially dopa, dopamine, their acylated products, side-chain dehydrogenated products, and molecules embedded with these structural elements, can lead to the development of novel biomaterials for future use. This review summarizes various aspects of catecholamine chemistry that will be useful for this purpose.

## 2. Common Biochemical Pathway of Catecholamines

**Figure 1:**
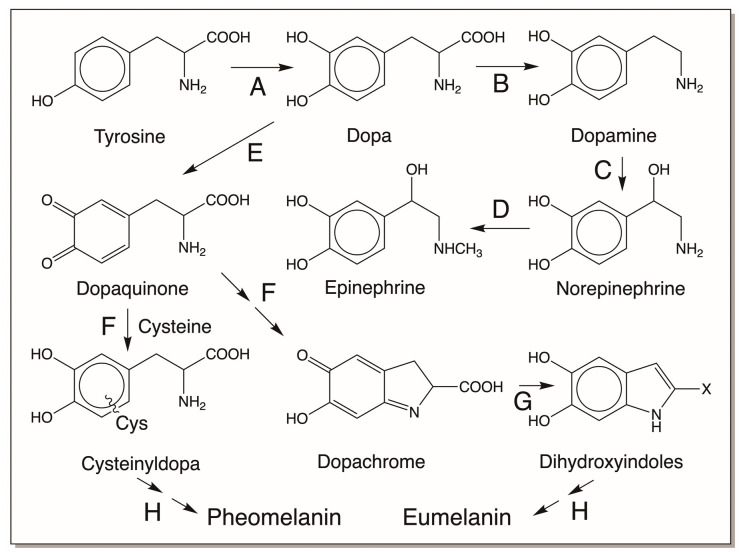
The common biochemical fate of dopa. Tyrosine is hydroxylated by tyrosine hydroxylase (A), generating dopa. Dopa decarboxylase (B) produces dopamine from dopa. Dopamine β-hydroxylase (C) converts dopamine to norepinephrine. Methylation of norepinephrine (D) yields the hormone epinephrine, also known as adrenaline. In the skin, hair, and eye of animals, phenolic pigment melanin is generated from dopa through oxidative polymerization reactions. Tyrosinase (E) oxidizes dopa to dopaquinone. It also has the capacity to convert tyrosine to dopaquinone (not shown in figure). Intramolecular cyclization of dopaquinone (F = nonenzymatic reaction) produces dopachrome which undergoes transformation to produce 5,6-dihydroxyindole (X = H) and/or 5,6-dihydroxyindole-2-carboxylic acid (X = COOH) by dopachrome converting enzymes (G). Oxidative polymerization (H) of dihydroxyindoles generates black to brown eumelanin pigments. On the other hand, oxidative polymerization (H) of cysteinyl dopa, formed by the addition of cysteine to dopaquinone (F = nonenzymatic reaction), produces yellow to red pheomelanin pigments.

Catecholamines are biosynthesized starting from the common amino acid tyrosine. Hydroxylation of tyrosine produces dopa, the first catecholamine, that serves as the precursor for several biologically and physiologically important biomolecules. Dopa decarboxylase produces another crucial catecholamine, dopamine. Hydroxylation of the side chain of dopamine yields norepinephrine, which upon methylation produces the hormone epinephrine, also known as adrenaline (Figure 1). Apart from these four key biochemical components that are ubiquitous in most living organisms, most organisms also produce different melanins that are derived from catecholamines. In animals, dopa undergoes oxidation to form dopaquinone. Intramolecular cyclization of dopaquinone produces dopachrome, which undergoes either decarboxylation and/or isomerization, yielding 5,6-dihydroxyindole (DHI) and/or 5,6-dihydroxyindole-2-carboxylic acid (DHICA) [1,2,3,4,5,6]. Oxidative polymerization of these dihydroxyindoles eventually produces brown to black eumelanin pigments. Dopaquinone also undergoes addition reaction with the amino acid cysteine, generating cysteinyl dopa, which produces yellow to red pheomelanin pigments after oxidative polymerization (Figure 1). These are the common and well-known fates of dopa that are often found in biochemistry textbooks. But in this article, we will examine, in addition, several other reactivities of catecholamines that are key to the production of unique biomaterials.

## 3. Catecholamines Destined to Become Biopolymers

### 3.1. Melanin Biopolymers

**Figure 2:**
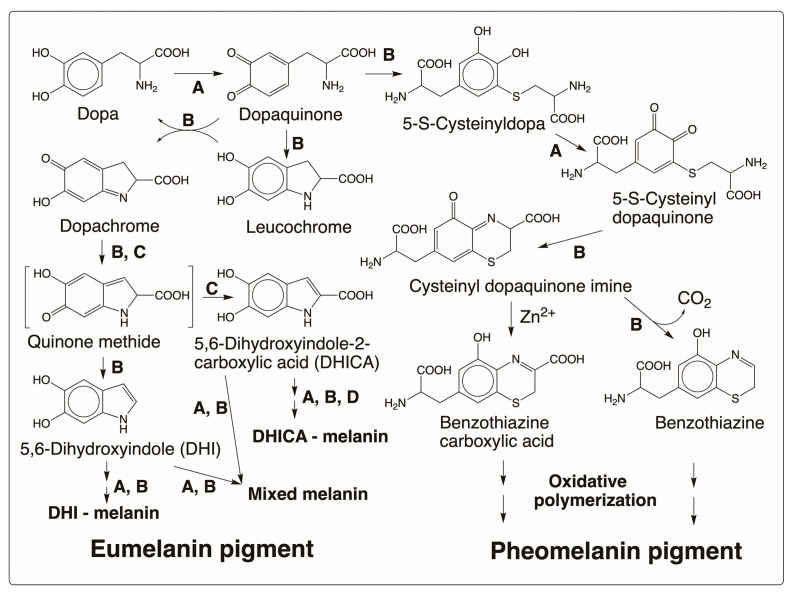
Biosynthesis of melanin pigment. Dopa formed from tyrosine (shown in Figure 1) is oxidized by tyrosinase (A) to dopaquinone. Dopaquinone undergoes nonenzymatic (B) cyclization and further oxidation to generate dopachrome. Dopachrome tautomerase (C) isomerizes dopachrome to 5,6-dihydroxyindole-2-carboxylic acid (DHICA) through a quinone methide intermediate. Dopachrome is also believed to undergo nonenzymatic decarboxylation through the quinone methide intermediate to form 5,6-dihydroxyindole (DHI). Oxidative polymerization of these two indoles produces brown to black eumelanin pigment (D = a specific DHICA oxidase). Initially formed dopaquinone can also be trapped by cysteine to produce cysteinyl dopa, which upon oxidation and further reaction can generate yellow to red pheomelanin pigment through nonenzymatic reactions, as shown.

Melanin is the phenolic biopolymer found in the skin, eye, and hair of all animals. Two types of melanin have been identified: yellow to red pheomelanin that arises by the oxidative polymerization of cysteinyl dopa and the brown to black eumelanin that arises from the oxidative polymerization of dihydroxyindoles. In addition, the existence of mixed melanin is also acknowledged. The melanin biosynthetic pathway has been extensively reviewed by several authors [1,2,3,4,5,6,7]. Therefore, it is not extensively discussed here and only a summary of the biosynthetic pathway is given. As shown in Figure 1, tyrosine is the precursor for melanin biosynthesis in all animals. It is converted to dopa by tyrosine hydroxylase, which then serves as the immediate precursor of melanogenesis. Tyrosinase oxidizes dopa to dopaquinone. It also has the capacity to convert tyrosine to dopaquinone. Dopaquinone thus formed undergoes intramolecular cyclization, generating leucochrome. Leucochrome undergoes double decomposition with dopaquinone, generating back dopa and producing dopachrome as the end product (Figure 2). Dopachrome tautomerase isolated from mammalian systems isomerizes dopachrome to DHICA probably via the transient quinone methide intermediate [5]. It is believed that DHI arises in the mammalian system through nonenzymatic reactions via the same quinone methide intermediate. A separate DHICA oxidase might be associated with the oxidation of DHICA [8], but most likely either tyrosinase and/or nonenzymatic oxidations initiate the oxidative polymerization of dihydroxyindoles to eventually produce brown to black eumelanin pigment in mammals. The yellow to red pheomelanin pigment arises by the oxidative polymerization of cysteinyl dopa formed by the condensation of cysteine with dopaquinone [1,2,3,4,5,6]. The major product of the reaction, 5-S-cysteinyldopa, is further oxidized by tyrosinase to its quinone. Cyclization of 5-S-cysteinyl dopaquinone generates cysteinyl dopaquinone imine product, which can either isomerize to benzothiazine carboxylic acid or decarboxylate to benzothiazine. Eventual oxidative polymerization of these benzothiazine derivatives will produce the pheomelanin pigment [1,2,3,4,5,6].

**Figure 3:**
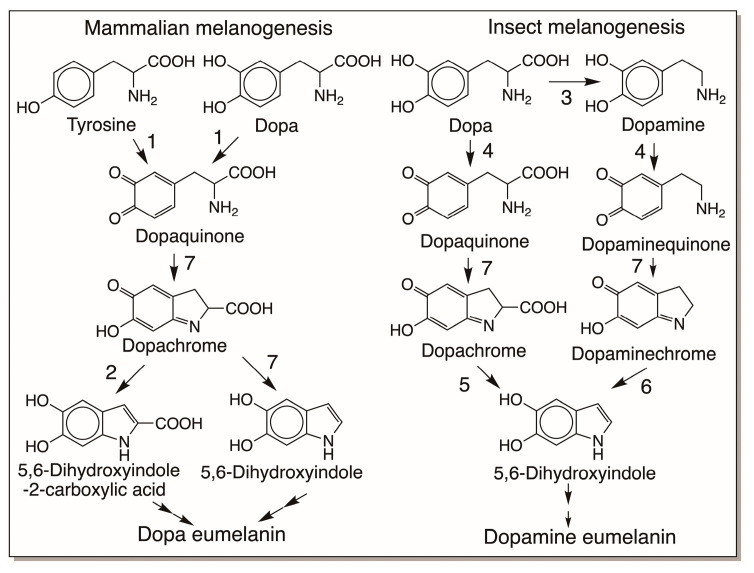
Comparative biochemistry of mammalian and insect eumelanogenesis. In mammals, both tyrosine and dopa are converted by tyrosinase (1) to dopaquinone. Dopaquinone undergoes intramolecular cyclization and oxidation through nonenzymatic reactions (7), generating dopachrome. A specific dopachrome tautomerase (2) converts dopachrome to 5,6-dihydroxyindole-2-carboxylic acid in mammals. Dopachrome is also nonenzymatically converted to 5,6-dihydroxyindole. Oxidative polymerization of these two dihydroxyindoles produces brown to black eumelanin in mammals. In insects, dopa is mostly decarboxylated by dopa decarboxylase (3) to produce dopamine. Phenoloxidase (4) oxidizes dopamine as well as any dopa to their corresponding quinones, which are nonenzymatically (7) converted to chromes. Insects possess a unique dopachrome decarboxylase/tautomerase (5) which converts any dopachrome formed in the system to 5,6-dihydroxyindole. A specific dopaminechrome tautomerase (6) converts dopaminechrome to 5,6-dihydroxyindole, which is oxidatively polymerized to give black-colored eumelanin pigment in insects. Thus, mammals produce dopa eumelanin, but insects produce mostly dopamine eumelanin. (Not shown in figure—Pheomelanin is mostly formed from dopa in mammals and from dopamine in insects).

Recent work indicates that insects are unique in that they produce mostly dopamine melanin and not dopa melanin, thus significantly differing from the mammalian melanogenic process (Figure 3) [7,9]. In practically all insects, dopamine and not dopa serves as the primary source for melanin production. Insects need large amounts of N-acyldopamine derivatives for the cuticular sclerotization reaction that is extremely vital for their survival [10,11,12,13,14,15,16]. They convert most of the available dopa to dopamine derivatives and store them. Since dopamine is not converted back to dopa, only dopamine serves as the melanin precursor for insects [7,9]. Also, both sclerotization and melanization reactions occur in insect cuticles, with sclerotization preceding melanization. For sclerotization reactions, most of the available N-acyldopamines are used up, the remaining material is hydrolyzed [17], and the resultant dopamine is diverted for melanization. Accordingly, chemical analysis of insect melanin reveals that they are mostly generated with dopamine as the precursor and not dopa [9]. Recent studies also confirm the existence of a separate dopaminechrome isomerase that participates in insect melanogenesis [18]. The scheme of reactions shown in Figure 3 illustrates the differences between mammalian melanogenesis and insect melanogenesis.

### 3.2. N-Acyldopamine Derivatives and Cuticular Sclerotization

**Figure 4:**
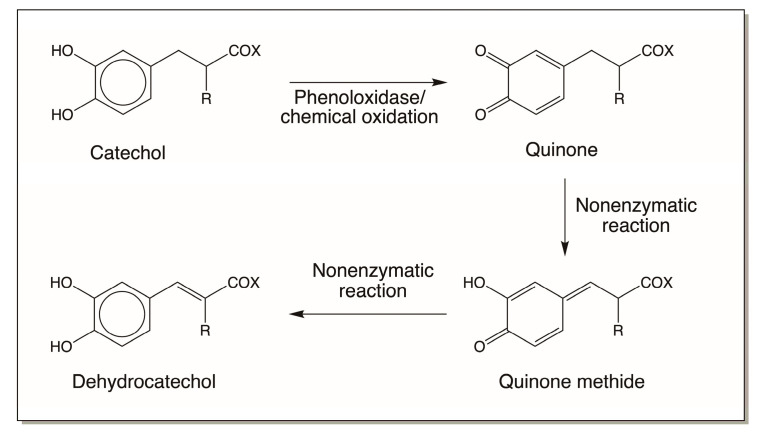
Spontaneous side-chain desaturation of some quinones. Both dihydrocaffeiyl methyl amide (R = H; X = NHCH_3_) and dihydrocaffeiyl methyl ester (R = H; X = OCH_3_), upon oxidation to their corresponding quinones, undergo rapid nonenzymatic conversion to produce side-chain dehydrogenated caffeic acid derivatives. Even peptidyl dopa derivatives (R = NHCOCH_3_, X = OCH_3_, or OCH_2_CH_3_) form dehydro dopa derivatives by a similar process.

The exoskeletons of all insects are hardened to protect their soft bodies by a process known as sclerotization [10,11,12,13,14,15,16]. In addition, other structures, such as oothecae, egg cases, egg sacs, chorions, and silk, produced in insects are also often hardened by the sclerotization process [19]. During sclerotization, catecholamine derivatives, in particular, N-acetyldopamine (NADA) and N-β-alanyldopamine (NBAD), are activated to form reactive intermediates that crosslink cuticular proteins and chitin, a carbohydrate-based structural polymer, to form the tough exoskeleton [10,11,12,13,14,15,16]. Dopa serves as the precursor for these two compounds. Dopa decarboxylase, as usual, produces the necessary dopamine by decarboxylation reaction. The resultant dopamine is acylated by appropriate enzymes to produce NADA and NBAD. While both NADA and NBAD can be oxidized to their corresponding quinones and the resultant quinones can be used to form quinone amino acid adducts, like cysteinyl dopa and cysteinyl dopamine, their full potential to form maximum crosslinks can only be achieved if their side chains can be desaturated [5,16]. This process is much like introducing the double bond in the side chains of dopa and dopamine when they form dihydroxyindoles (see Figure 2). Again, the process requires the conversion of quinone first to its quinone methide analog and then another isomerization to produce the side-chain desaturated compound [5]. Side-chain desaturation can be spontaneous in some cases. For example, both dihydrocaffeiyl amide and dihydrocaffeiyl ethyl ester (Figure 4), upon oxidation to their corresponding quinones, exhibit spontaneous nonenzymatic isomerization, generating their side-chain desaturated compounds via transient quinone methide intermediates [5,15,16]. The peptidyl model compounds N-acetyl dopa methyl ester as well as N-acetyl dopa ethyl ester also exhibit the same reaction sequence, producing their corresponding dehydro dopa derivatives (Figure 4) [6,16,20,21].

**Figure 5:**
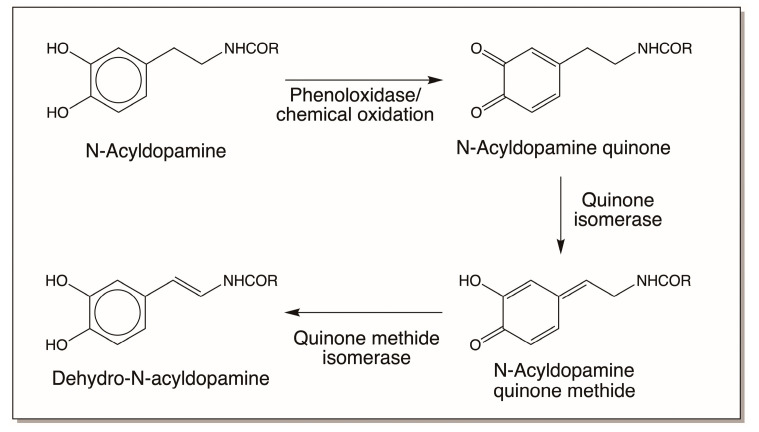
Enzyme-catalyzed side-chain desaturation of N-acyldopamines. Unlike dihydrocaffeiyl derivatives which exhibit spontaneous nonenzymatic side-chain desaturation, the introduction of double bonds in both N-acetyldopamine and N-β-alanyldopamine calls for the use of a three-enzyme system comprised of phenoloxidase as the initial oxidant and two isomerases, quinone isomerase and quinone methide isomerase, without which side-chain desaturation of these compounds cannot be achieved (R = CH_3_ for N-acetyldopamine; CH_2_CH_2_NH_2_ for N-β-alanyldopamine).

Such spontaneous isomerization reactions are not possible for simple N-acyldopamine derivatives, and enzymatic assistance is needed to introduce the double bonds in their side chains (Figure 5). The first reaction, *viz*., quinone-to-quinone methide isomerization, occurs spontaneously and not enzymatically in many cases [5,16], but for N-acyldopamine quinones an enzyme is needed to cause this conversion [16]. Quinone isomerase converts a few 4-alkyl-*o*-quinones to isomeric 2-hydroxy-*p*-quinone methides by tautomerization reaction. Thus, both N-acetyldopamine quinone and N-β-alanyldopamine quinone are converted to their corresponding quinone methides by this enzyme. The resultant quinone methide largely undergoes water addition to form side-chain hydroxylated products [16]. However, when the next enzyme, quinone methide isomerase, is available, the quinone methides are converted to dehydrodopamine derivatives, thus introducing the double bond in the side chain of N-acyldopamine derivatives [16]. In this context, it is important to draw particular attention to the presence of many small peptidyl dehydro dopa derivatives found in marine organisms, which are summarized in the next section.

### 3.3. Tunichromes and Related Marine Compounds

Tunicates, also known as ascidians, are sessile marine filter-feeding invertebrates. Several species of tunicates accumulate significant amounts of catecholamine peptides in their blood cells [22,23,24,25,26,27,28,29]. Since many tunicates also accumulate metal ions, such as vanadium and iron, in their blood cells, it is often misunderstood that the catecholamine peptides are somehow associated with metal ion chelation and transport. However, the association of these two groups of materials in different cell types caused serious doubt about this proposal [26]. Possible functions of tunichromes in the biochemistry and physiology of truncates have been extensively discussed and summarized in ref. [26]. Some of them are known to possess antibiotic and antioxidant properties [28,29]. The current understanding is that these catecholamine derivatives are associated with innate immune response (defense reaction), tunic (exoskeleton) formation, and wound healing [26].

**Figure 6:**
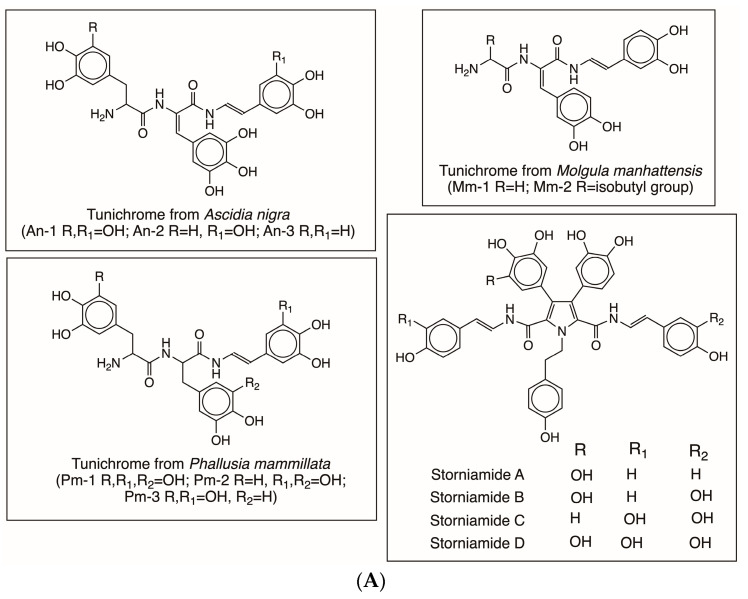

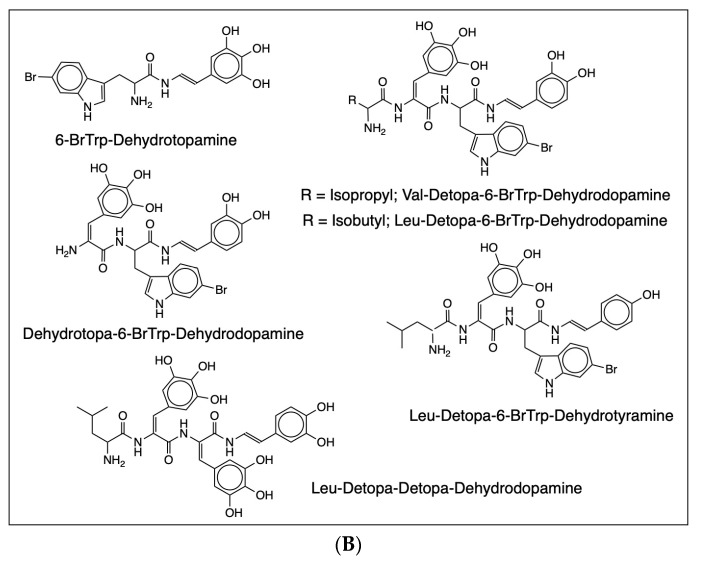
(**A**) Structures of tunichromes, and storniamides. The chemical structures of tunichromes and storniamides listed in Table 1 are given here. (**B**) Structures of clionamide and celenamides. The chemical structures of clionamide (6-BrTrp-Dehydrotopamine), Celenamide A (Leu-Dehydrotopa-6-BrTrp-Dehydrodopamine), Celenamide B (Val-Dehydrotopa-6-BrTrp-Dehydrodopamine), Celenamide C (Leu-Dehydrotopa-6-BrTrp-Dehydrotyramine), Celenamide D (Leu-Dehydrotopa-Dehydrotopa-Dehydrodopamine), and Celenamide E (Dehydrotopa-6-BrTrp-Dehydrodopamine) listed in Table 1 are given here.

Tunichromes An-1, An-2, and An-3 were the first group of compounds isolated from the tunicate *Ascidia nigra* [22,23,30,31]. Subsequently related compounds were isolated from different species of tunicates, such as *Molgula manhattensis*, *Phallusia mammillata*, and *Styela plicata* [22,23,24,25,26,27,28]. The compound names and chemical names of several of these compounds are listed in Table 1. The structures of several of these compounds are illustrated in Figure 6A,B.

A cursory glance at the table and the associated Figure 6A,B clearly illustrates that these peptide units possess a common structure, namely, a dehydrodopa skeleton. This, combined with the fact that sea water is alkaline, introduces new possibilities of nonenzymatic and free-radical-mediated reactions, as will be discussed later.

### 3.4. Peptidyl Dopa Derivatives

**Figure 7:**
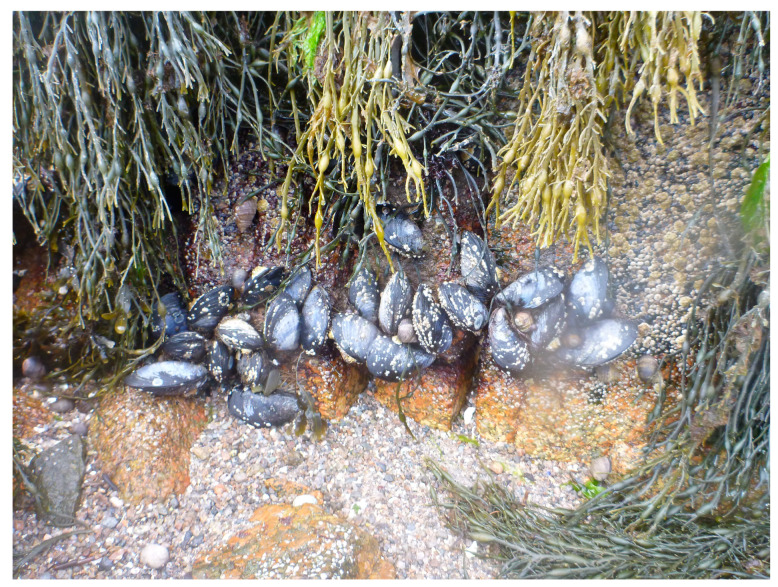
Marine mussels clinging to solid surface. Mussels and other marine organisms cling to solid surfaces through their feet, using powerful adhesive proteins that are known to contain dopa units.

Marine mussels often cling to rocks and other solid surfaces through a powerful adhesive which works even in the presence of water (Figure 7). It allows the mussels to resist dislodging by powerful waves and sea currents. The cause of this powerful adhesion is attributed to the mussel foot protein that contains abundant amounts of dopa. Waite and his associates pioneered the studies on this novel adhesive system [32,33,34,35,36]. This system obviously calls for the interaction of biomaterials with inorganic solid surfaces. Catecholamine derivatives associated with this process are well known for metal chelating properties that play a crucial role in adhesive properties of the mussel foot fibers [32,33,34,35,36,37,38,39]. Among all the catecholamine biomaterials, these proteins and similar molecules seem to offer unique avenues to develop novel bioadhesives with wet/dry adhesive properties [37,38,39,40,41].

## 4. Chemical Reactivities of Catechols

From the foregoing summary, it is evident that catecholamines are used for a variety of purposes. To achieve such properties, catecholamines use a variety of mechanisms. These include and are not limited to metal chelation, free radical production, quinone formation, quinone methide formation, and dehydro dopa formation. The normal interactions, such as hydrogen bonding, hydrophobic interaction, and other types of interactions, will not be considered here, although they also contribute significantly and sometimes monumentally to the binding ability of catechols. In the following section, some of these mechanisms are examined.

### 4.1. Reactivity Exhibited through Quinone Formation

**Figure 8:**
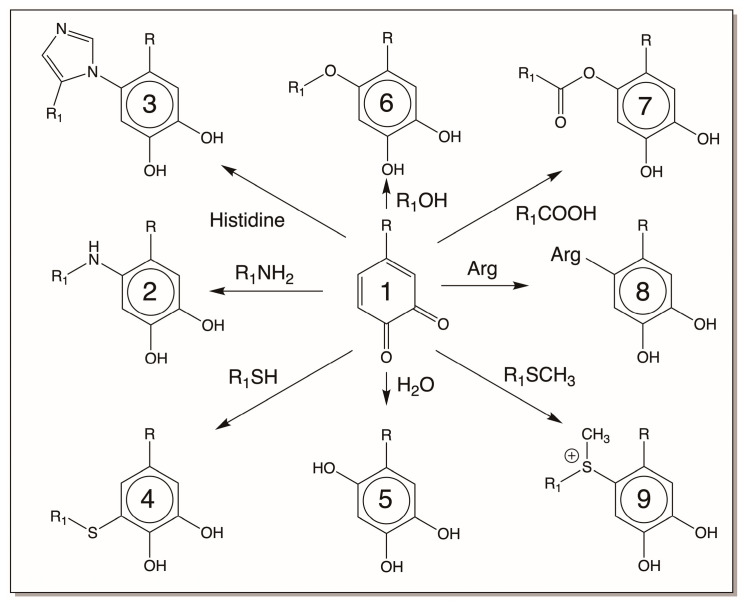
Reactivities of *o*-quinone. *o*-Quinones typically undergo nucleophilic addition reactions with various available nucleophiles present on proteins and other macromolecules. Primary amines, secondary amines, hydroxyl groups, and carboxyl groups produce the typical Michael-1,4-addition adducts. Even water can add on to *o*-quinones, producing hydroxy catechols. Thiols seems to add on to quinones by free-radical coupling reactions, while thiol esters and methionine may react by normal nucleophilic addition reactions. R = side chain of catechol; R_1_ = different side chains. (Compound 1 = parent quinone; 2 = quinone amine adduct; 3 = quinone histidine adduct; 4 = quinone thiol adduct; 5 = quinone water adduct; 6 = quinone ether adduct; 7 = quinone ester adduct; 8 = quinone arginine adduct; 9 = quinone methionine adduct).

**Figure 9:**
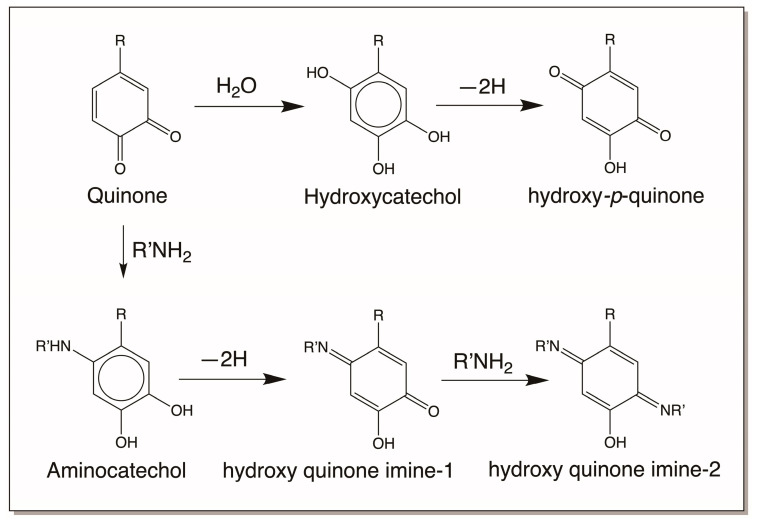
Further reactions of hydroxy catechols and amino catechols. Both hydroxy catechol formed by the addition of water to quinone and amino catechol formed by the addition of amine to quinone readily undergo aerial oxidation (or oxidation by parent quinones) to hydroxy-*p*-quinone and hydroxyquinone imine, respectively. Hydroxy quinone imine also undergoes further Schiff base formation with amine, generating quinone imines (R = side chain of catechol; R’ = side chain of amine) [44,50].

The first reactive intermediate that is widely recognized for catechols is its two-electron oxidation product, quinone. *o*-Diphenols upon enzymatic and even nonenzymatic oxidation generate their corresponding *o*-quinone. Nature has a variety of enzymes to produce quinones [3,4,5,6,7,12,13,14,15,16]. As mentioned in Section 2, tyrosinase readily oxidizes catechols to quinones [3,4,5,6,7]. In insects and other arthropods, genetically different but functionally similar enzymes that are generally referred to as phenoloxidases perform this reaction [13,14,15,16]. Both *o*-diphenol oxidase and *p*-diphenol oxidase (laccase) of insect origin could oxidize *o*-diphenols to their corresponding quinone derivatives [13,14,15,16]. In addition, peroxidases also assist in the production of quinones from *o*-diphenols but by an indirect mechanism. They primarily oxidize catechols to their semiquinone radicals, which undergo nonenzymatic dismutation to regenerate the parent catechol and *o*-quinone product [42,43]. Some quinones also generate quinones from certain catechols nonenzymatically by oxidation. For example, dopaquinone oxidizes leucochrome to dopachrome. During this process, it gets reduced to dopa (Figure 2). Regardless of the way they are formed, *o*-quinones are highly reactive electrophiles and seek nucleophilic addition reactions (Figure 8). We have already seen two key reactions of quinone in melanin biosynthesis, *viz*., cysteine addition to dopaquinone and intramolecular cyclization of dopaquinone (Figure 2). Primary amines react rapidly with quinones, forming amino catechols [5,16]. Thus, intramolecular cyclization of dopaquinone produces leucodopachrome [1,2,3,4,5,6,7] (Figure 2). External addition of primary amine nucleophiles, as shown in Figure 8, will produce amino catechols. These amino catechols are susceptible to nonenzymatic oxidation most readily. Such reactions will produce aminoquinones, which can further exhibit reactions forming quinone imines (Figure 9). Such products have been identified in many systems and are now known to be part of enzymatic active sites [44]. Secondary amines such as the imidazole group of histidine also exhibit addition reactions (Figure 8) [45,46,47,48,49]. It is likely that even arginine can add to catechols. These reactions yield typical Michael-1,4-addition products (Figure 8). Hydroxyl groups can function as nucleophiles and produce hydroxy catechols which will suffer nonenzymatic oxidation, forming hydroxy-*p*-quinones (Figure 8 and Figure 9) [50]. Finally, the carboxyl group can also add on to the quinone nucleus, producing ester derivatives [5]. While these reactions all occur via Michael-1,4-addition reactions, thiols seem to violate this normal nucleophilic addition. Thiols, which can react even faster than amines, do not produce the normally expected Michael-1,4-adducts, as they do not seem to add via normal nucleophilic reactions [1,2,3,4,5,6,7,51,52,53]. Instead, the coupling of dopaquinone and cysteine produces 5-cysteinyldopa as the major product [1,2,3,4,5,6,7,53]. Different theories have been proposed for this abnormal addition reaction, but recently it has been conclusively proven to be a free-radical coupling reaction [53]. On the other hand, methionine seems to produce the normally expected Michael-1,4-adduct only [54,55].

### 4.2. Reactivity Exhibited through Quinone Methide Formation

**Figure 10:**
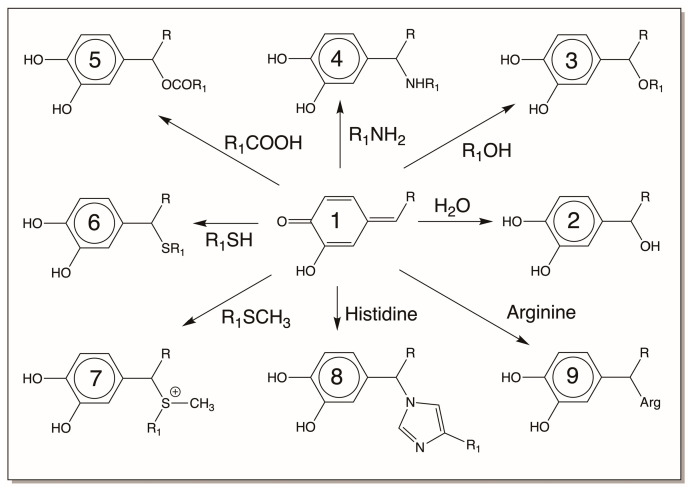
Reactivities of *p*-quinone methide. *p*-Quinone methides undergo nucleophilic addition reactions at 1,6-position, generating side-chain substituted products. R = side chain of catecholamine derivatives; R_1_ = different side chains. (Compound 1 = parent quinone methide; 2 = quinone methide water adduct; 3 = quinone methide ether adduct; 4 = quinone methide amine adduct; 5 = quinone methide ester adduct; 6 = quinone methide thiol adduct; 7 = quinone methide methionine adduct; 8 = quinone methide histidine adduct; 9 = quinone methide arginine adduct.)

The next key reactive metabolite associated with various catecholamine derivatives is the *p*-quinone methide. Formation of this intermediate is possible only for 4-alkylcatechols and related compounds. In the case of 4-alkylcatechols, their two-electron oxidation products, quinones, can isomerize to tautomeric hydroxy-*p*-quinone methides [5,15,16]. This reaction could be nonenzymatic in some cases (Figure 4) or require an enzyme is some other cases (Figure 5). Quinone isomerase isolated from a number of insects catalyzes the conversion of 4-alkylquinones to *p*-quinone methides [16]. This reaction is absolutely essential for sclerotization of the insect cuticle [12,13,14,15,16]. Regardless of the mode of formation, the resultant quinone methides are highly reactive. The quinonoid portion tries to aromatize, which results in the nucleophilic addition of various electrophiles at 1,6-positions. Thus, they yield side-chain substituted products, as shown in Figure 10. Practically all nucleophiles add on to quinone methides, generating side-chain substituted compounds.

### 4.3. Reactivity Exhibited through Dehydrodopa Formation

**Figure 11:**
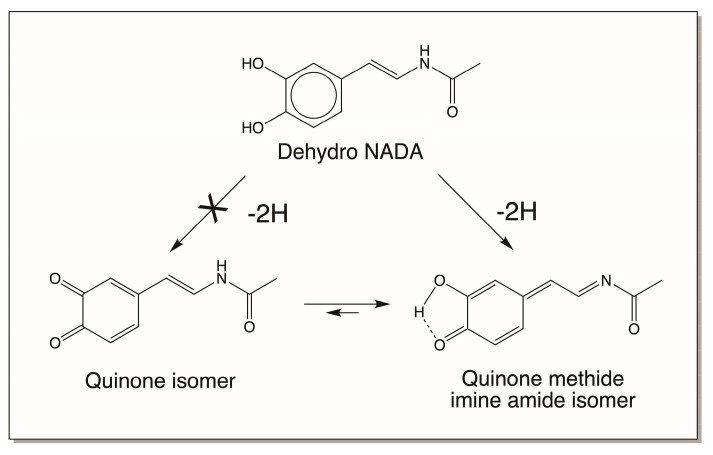
Oxidation of dehydro NADA under physiological conditions yields, not the normally expected quinone, but its isomeric quinone methide imine amide.

**Figure 12:**
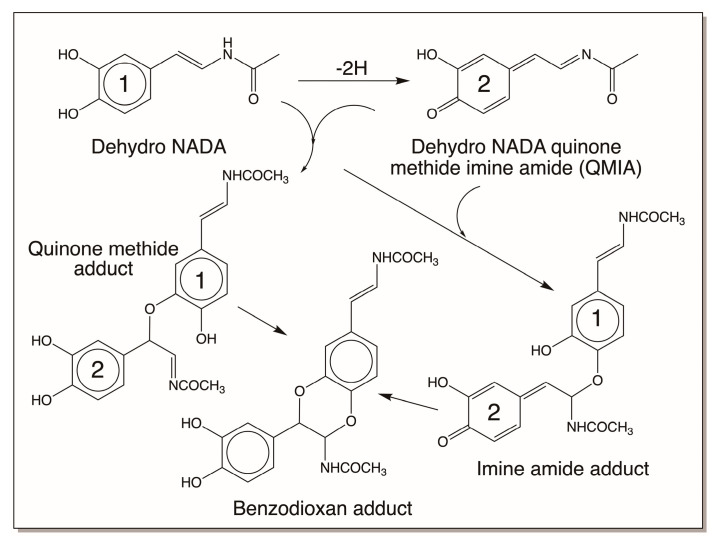
Oxidation of dehydro NADA under physiological conditions yields QMIA, which adds on to its side chain both the hydroxyl groups of the parent catechol, resulting in the formation of a benzodioxan dimer. The dimers then add on to QMIA, producing trimers and other oligomers (not shown in figure). (1 = parent dehydro NADA; 2 = QMIA portion.)

As shown in Figure 10, quinone methides also behave much like quinones in exhibiting nucleophilic addition reactions, except for the fact that the additions occur at the side chain and not in the ring. But one of the reactions of a quinone methide that is not possible for quinones is the isomerization to a dehydro derivative if an electron withdrawaling group is present on the beta carbon atom. Thus, as explained in Section 3.2, quinone methides of both dihydrocaffeiyl methyl amide and dihydrocaffeiyl methyl ester spontaneously and rapidly exhibit tautomerization to produce side-chain desaturated caffeic acid derivatives (Figure 4) [5,20,21]. However, when the CO and NH groups in dihydrocaffeiyl methyl amide and related compounds are interchanged, spontaneous tautomerization is not possible and enzymatic intervention is necessary to generate the side-chain desaturated catechols (details outlined in Section 3.2) [16]. The oxidation of these compounds uniquely produces a different reactive intermediate called quinone methide imine amide (QMIA), which has the capability to exhibit addition reactions through both its side-chain carbon atoms. See Ref. [16] for a detailed review. The first reaction witnessed in this case is the benzodioxan formation from the oxidation product of dehydro NADA. Upon enzymatic (or even nonenzymatic) oxidation under physiological conditions, the immediate product formed in this case is the QMIA and not the normally expected quinone product (Figure 11). The conventional quinone is formed only when dehydro NADA is oxidized under acidic conditions. Even raising the pH of the reaction to near-neutral conditions converts the quinone rapidly to the more stable QMIA isomer. The quinone-to-quinone methide isomerization is a base-catalyzed reaction [56,57] and hence at near-neutral and alkaline conditions the QMIA is the observable product and not the quinone. Therefore, reactions associated with dehydro NADA at physiological pH are manifested by the QMIA and not the quinone [16]. Semiquantitative calculations show that the QMIA is more stable than the quinone by about 6 kcal/mole [16]. The extra hydrogen bonding that occurs in QMIA, as shown in Figure 11, may provide additional stabilization. QMIA has two reactive sites. The quinone methide nucleus undergoes Michael-1,6-addition reaction, regenerating the aromatic ring. The side-chain imine amide part undergoes addition, resulting in the saturation of the original double bond. Since the two phenolic groups of parent dehydro NADA are stereochemically well suited to undergo addition at both the sites, it rapidly generates benzodioxan dimers, as shown in Figure 12. Such addition reactions indicate that other nucleophiles can also add on to both the side-chain carbon atoms of QMIA, forming adducts and crosslinks [58].

**Figure 13:**
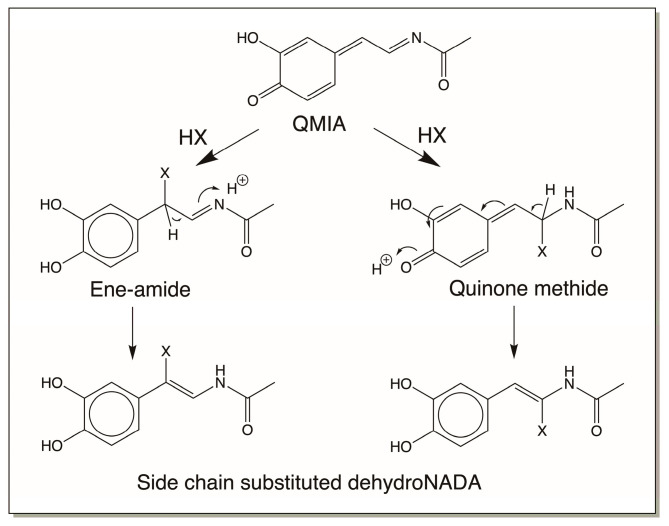
Dehydro NADA QMIA can also undergo substitution reaction, regenerating the side-chain double bond. The regenerated dehydro compound can undergo further oxidation to another QMIA intermediate that can also react with nucleophiles (not shown in figure).

Examination of the reactions of dehydro NADA resulted in some unusual findings. First, the QMIA formed undergoes, not only addition reactions, but also substitution reactions [58,59]. In two instances, regeneration of the side-chain double bond following substitution at the side chain has been reported, as shown in Figure 13 [16,58,59]. This presents an interesting scenario for dehydro NADA and related compounds. (A) They undergo substitution with available external nucleophiles, and (B) they still can exhibit further oxidation to substituted QMIA and add on to more nucleophiles. Second, the benzodioxan formation can occur not only through QMIA intermediate formation, but also by two additional mechanisms.

**Figure 14:**
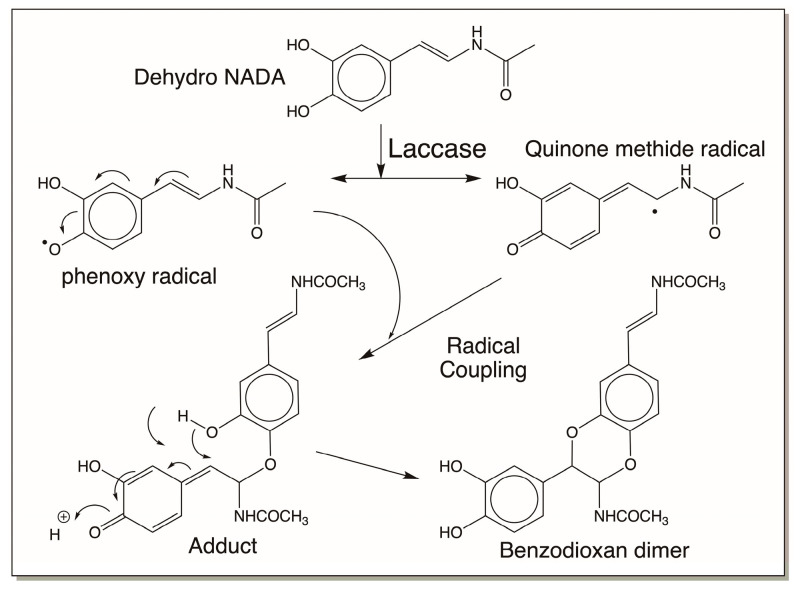
Laccase-catalyzed oxidation of dehydro NADA. Oxidation of dehydro NADA by laccase produces semiquinone free radicals which couple immediately, forming benzodioxan dimers. In this case, trimers and other oligomers are not witnessed.

In the first mechanism, dehydro NADA oxidation by laccase causes semiquinone radical production that results in coupling, providing the benzodioxan dimer only, as shown in Figure 14 [16]. This is contrary to the reaction that occurs with tyrosinase, where dimers can go on adding another molecule of QMIA, forming trimers and other oligomers [16].

**Figure 15:**
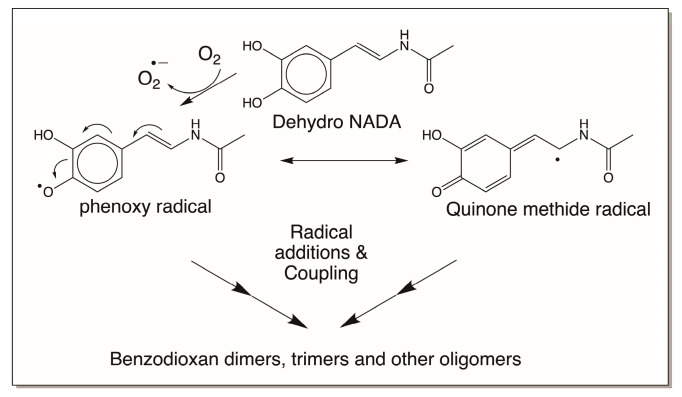
Aerial oxidation of dehydro NADA under mild alkaline conditions. Under mild alkaline conditions, dehydro NADA reacts rapidly with molecular oxygen, producing free radicals and superoxide anions. Radical coupling and addition results in polymerization reaction. Therefore, in this case, trimerization and other oligomerization reactions are also witnessed.

In the second mechanism, which also involves free radical formation, dehydro NADA exhibits polymerization reactions [16]. Dehydro NADA and related compounds, such as tunichromes and others, are extremely sensitive to air and pH. For example, tunichromes are so unstable that their synthesis requires protection of the hydroxy groups. Upon removing the protecting groups during the final stage of the synthesis, the tunichromes become so unstable and rapidly undergo polymerization reactions [22,23,24,25,26]. Dehydro NADA itself is reasonably stable in a solid state under an argon atmosphere, but upon exposure to air it exhibits slow aerial oxidation. In solution, it is stable under acidic and near-neutral conditions, but as soon as the pH is raised, even to about 7.5, it becomes sensitive to oxygen. The interaction of dehydro NADA with molecular oxygen produces semiquinone radicals and superoxide anions. Continuous production of free radicals and coupling causes dimerization as well as trimerization reactions. Note that even though laccase reaction also produces free radicals, they do not produce superoxide anions, which causes continuous production of free radicals and leads to polymerization reactions (Figure 15).

**Figure 16:**
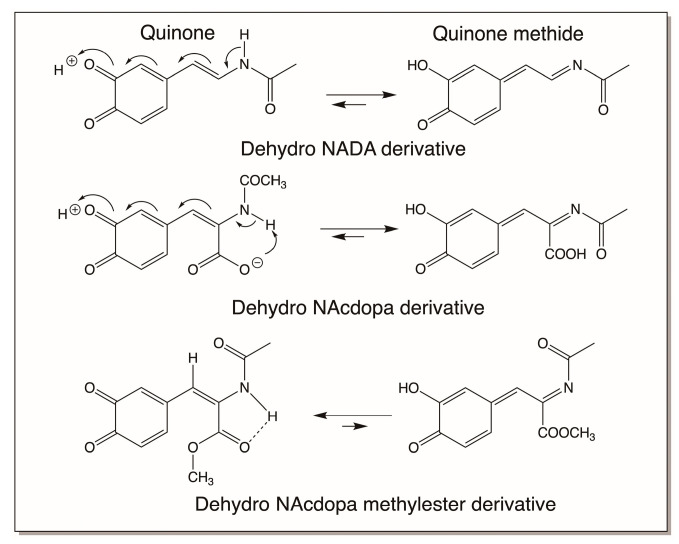
Differential stability of quinone versus quinone methides. In the case of both dehydro NADA and dehydro N-acetyl dopa, the quinone methides are more stable than the corresponding quinones. The opposite happens with dehydro N-acetyl dopa methyl ester, where the quinone is more stable than the quinone methide.

Finally, one must be aware of the fact that not all dehydro compounds will generate stable quinone methide derivatives. There are instances in which the conventional quinones could be stable products as well. Thus, while both dehydro NADA and dehydro N-acetyl dopa produce more stable quinone methide derivatives, as shown in Figure 16, dehydro N-acetyl dopa methyl ester produces only the normal quinone as the stable product [16]. This is because of the differential stabilization of quinone versus quinone methide isomers. Therefore, in case of peptidyl dehydrodopa derivatives, which are more similar to dehydro N-acetyl dopa methyl esters, quinones will be the dominant oxidation species.

**Figure 17:**
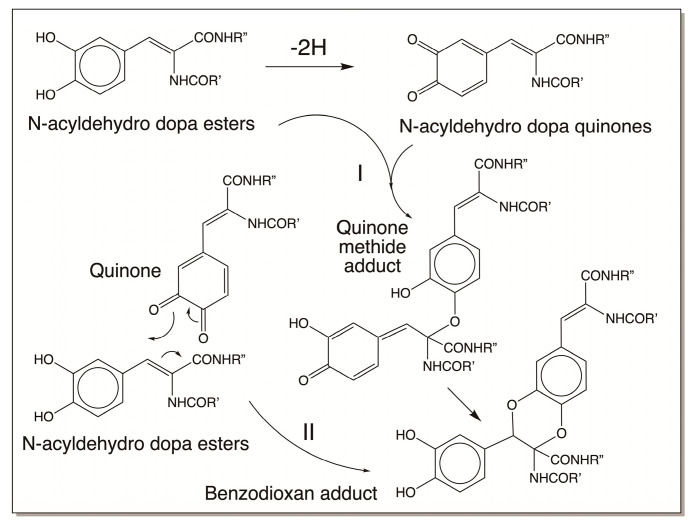
Diels–Alder-type addition reactions that are possible for dehydro N-acetyl dopa esters. Upon oxidation, N-acyldehydro dopa esters produce stable quinones that can undergo ionic Diels–Alder-type addition to form benzodioxan adducts.

Interestingly, the dehydro dopa quinone ester also exhibits dimerization reactions, not through the quinone methide isomer, but by itself through a different mechanism involving ionic Diels–Alder-type addition (Figure 17). Two such cases have been identified in recent years where side-chain desaturated catechols have been shown to exhibit benzodioxan-type adduct formation by this mechanism [60,61]. Thus, irrespective of quinone, quinone methide, or free radical intermediacy, side-chain dehydrogenated catecholamines can exhibit dimerization and benzodioxan formation.

### 4.4. Reactivity Exhibited through Free Radicals

**Figure 18:**
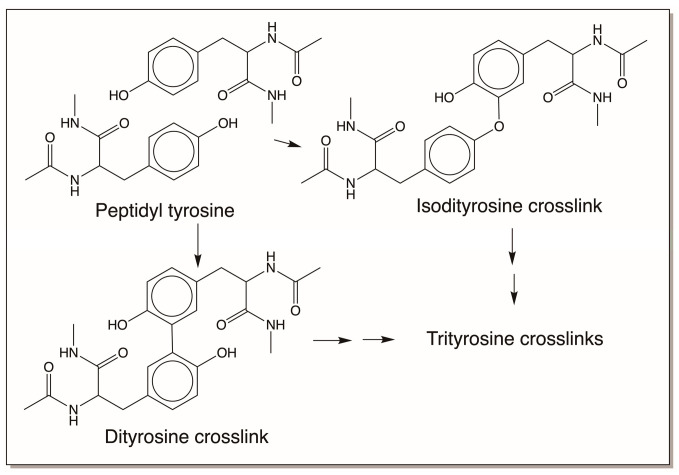
Peroxidase-mediated crosslinking of the tyrosine-rich protein resilin results in dityrosine and trityrosine crosslink formation. The figure also includes the production of isodityrosine crosslinks that are yet to be identified in insects.

Simple *o*-diphenolic compounds do not spontaneously produce free radicals. However, some of the compounds are oxidized by enzymes such as peroxidase that could lead to free radical production. The free radicals thus formed will exhibit a variety of reactions. We have already seen the free radical formation from dehydro NADA. Dehydro NADA and its derivatives are extremely air-sensitive and even at slightly alkaline conditions undergo rapid oxidation to form free radicals. The free radicals, such as phenoxy radicals and quinone methide radicals, that are formed during the reaction also seem to undergo coupling to produce benzodioxan-type dimers and related oligomers. But other adduct formations are also possible for free radicals. Peptidyl tyrosine residues in the protein resilin present in the elastic cuticles of insects undergo peroxidase-mediated dityrosine formation. See Ref. [16] for a detailed review. Similar adduct formation has been noticed in other systems as well [62]. Both trityrosine formation as well as isodityrosine formation are also possible (Figure 18). Trityrosine has been isolated from the protein hydrolysates. But isodityrosine is yet to be identified in any insect systems.

**Figure 19:**
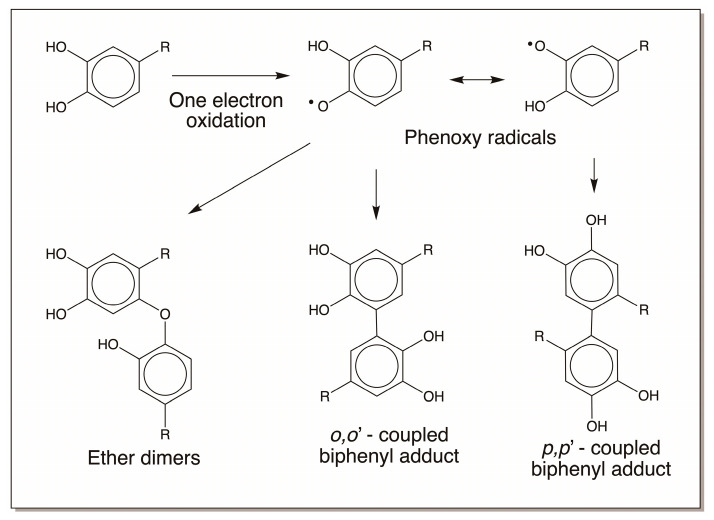
Peroxidase-mediated crosslinking of dopa derivatives can produce different dimeric products, as shown in the figure. Production of ether-type adducts, as well as different biphenyl dimers, is also possible.

Similar crosslink formation with peptidyl dopa derivatives is also highly likely both enzymatically and nonenzymatically. If oxidative enzymes such as peroxidases are present at the site, these catechols can be easily oxidized to free radicals. The resultant free radicals upon coupling will produce different kinds of dimeric products, such as *o,o’* coupled and *p,p’*-coupled (as well as *o,p*-coupled; not shown in figure) biphenyls and ether dimers, as shown in Figure 19. During this process, oxygen is reduced to superoxide anion, and it can oxidize another molecule of catechol to semiquinone and then reduce to hydrogen peroxide. However, enzymatic oxidation can lead to the production of free radicals. Unstable catechols can easily undergo nonenzymatic oxidation, producing the same products as well.

### 4.5. Catechols and Their Derivatives as Metal Chelators

**Figure 20:**
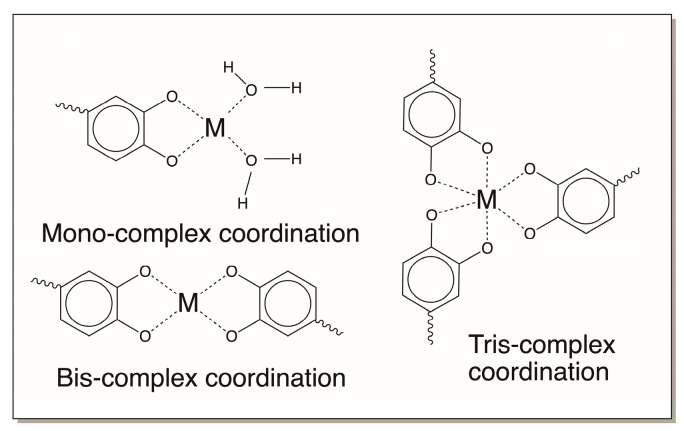
Complex formation of catechols with metal ions can result in mono-, bis-, and tris-coordinated metal catechol complexes.

Finally, catechols also exhibit excellent complexation reactions with metal ions [63,64]. The *o*-dihydroxy group is especially suitable for complex formation and provides a bidentate ligand to complex with a variety of divalent and polyvalent metal ions. Such interactions will produce very strong metal complexes. Mono-, bis-, and tris-coordination of catechols to various metal ions has been well documented in the inorganic chemistry literature [64]. The structures of some of these complexes are shown in Figure 20.

**Figure 21:**
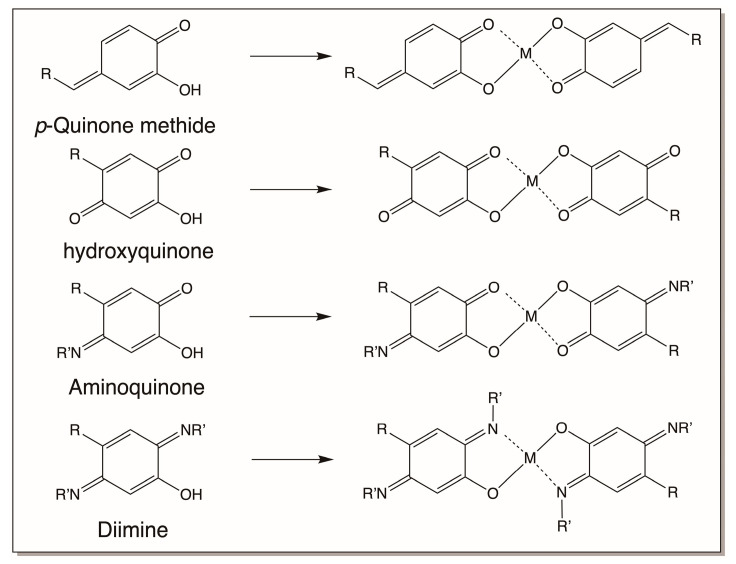
Complexation of metal ions by *p*-quinone methides, hydroxyquinones, amino quinones, and diimines. Only bis complex formation is shown in the figure.

Interestingly, several of the oxidation products of catecholamine derivatives, such as quinone methides, hydroxyquinones, aminoquinones, and diimines, can also complex with metal ions, as shown in Figure 21. Only bis complexes are shown in the figure. But mono as well as tris complexes are also possible for these compounds too. These complexation reactions will allow the entrapment of metal ions in the biomaterial to provide additional strength.

## 5. Catecholamine-Based Biomaterials

From the above summary, it is evident that catecholamines exhibit a wide variety of reactivities both through their aromatic rings and through their side chains. Time and again various groups have summarized the reactivities of catecholamines [1,2,3,4,5,6,7,22,23,24,25,26,32,33,34,35,36,37,38,63]. Since life evolved in the sea first, before terrestrial animals appeared, it is appropriate to consider what is going on in the sea animals first. Obviously to work under water (or in the presence of large amounts of water), the gluing and crosslinking process must use materials that are not easily washed off. Under such constraints, a protein-based glue that can form a polymer like adhesive is ideal. Accordingly, mussels have developed peptidyl dopa, a protein-based glue to bind to solid substrata [32,33,34,35,36,37,38,39,40,41]. Tunicates and other sea animals developed peptidyl dehydro dopas and dehydro dopas, which have been shown to be extremely reactive and “sticky”. They bind to solid surfaces such as glass with great ease and are extremely difficult to wash off [22,23,24,25,26,30,31]. Terrestrial insects, on the other hand, having limited use for the adhesive properties of these proteins, developed the use of water-soluble N-acyldopamine derivatives, NADA and NBAD, as crosslinking materials [10,11,12,13,14,15,16]. In this section, we will examine how these chemicals are used in making different biomaterials.

### 5.1. Peptidyl Dopa-Derived Biomaterial

As shown in Figure 7, marine mussels cling to rocks and other solid surfaces through a powerful adhesive which works even in the presence of water. It allows the mussels to resist being dislodged by powerful waves and sea currents. The byssal proteins in the foot of the mussel contain abundant amounts of dopa, which is considered the critical component responsible for providing adhesive properties. Several research groups have examined the properties and function of the byssal proteinaceous material with the aim of providing avenues to develop novel biomaterial adhesives that are nontoxic and can work under wet conditions. Such materials could be used for medical sutures, dental bonding, and other purposes where the gluing needs to take place in a water-rich environment.

The adhesion of a mussel to a wet, rough, and slimy surface is a carefully orchestrated process. The mussel foot creates an insulated reaction chamber, forming a vacuum that stimulates the deposition of adhesion proteins through the byssal threads [65]. Proton and electron pumps serve to lower the pH and control the redox environment [66]. It is thought that these conditions enable adhesive proteins to undergo controlled fluid–fluid phase separation, stimulating spreading and surface absorption [67]. These coacervates are denser than water, so they can be applied to the surface without significant dilution. The major byssal proteins, Mfp-1, Mfp-2, Mfp-3, Mfp-4, Mfp-5, and Mfp-6, are highly localized and have specific post-translational modifications, most importantly, the hydroxylation of tyrosine to form dopa [32,33,34,35,36,37,38,39,62]. It is hypothesized that the low pH acts to clean the surface and kill any attached microbes to prepare for surface attachment [32,33,34,35,36,37,38,39]. The reducing environment prevents premature oxidation of the dopa units of the byssal proteins. A combination of oxide formation with catechols, electrostatic interactions with amines and phosphates, hydrophobic interactions via phenylalanine, and coordination of catechols with metal ions on the surface all contribute to surface adhesion [32,33,34,35,36,37,38,39]. Once the foot detaches from the surface, the pH increases. Several of these surface interactions, such as electrostatic interactions and coordination with surface metal ions, strengthen as the pH increases. One could argue that oxidation of the catechols would likely weaken this surface interaction but there is some evidence that the reducing environment near the surface remains, even after detachment [68], and it is also speculated that the high cysteine content of Mfp-6, one of the main byssal proteins, helps to maintain the reducing environment of this surface layer [69]. Furthermore, the oxidation of catechols to quinonoid molecules can still afford metal ion chelation, as various oxidized derivatives will contribute to metal chelation [70,71,72,73,74,75].

Upon lift off, the pH increases at the reaction site (the pH of seawater is around 8.0). Catechols in the bulk of the proteinaceous deposit are oxidized, both enzymatically and nonenzymatically (due to the alkaline pH of the sea water) and initiate polymerization [32,33,34,35,36,37,38,39,74]. Polymerization can take place through several mechanisms: by the addition of amino acids on one byssal protein to the catechol ring of another byssal protein (Figure 8), or by didopa formation and by oxidative conversion to a dehydro dopa unit (Figure 4). The quinone methides formed during this process can form adducts with proteins and other materials (Figure 10). The dehydro dopa units also upon oxidation to QMIA will crosslink amino acid groups on other proteins across the double bond. The coupling of dopa units to form biphenyl and ether dimers (Figure 19) will contribute elastic properties to the fibrous deposit. Finally, the catecholic group and its oxidation products can form coordination bonds to Fe^3+^ and V^4+^ (Figure 20 and Figure 21). The Fe^3+^ and V^4+^ are accumulated by mussels and stored in the foot in vesicles separate from those of the byssal proteins [69,70,71,72,73,74,75]. The strength of a coordinate covalent bond between Fe^3+^ and a catechol is approximately one-quarter as strong as a covalent bond [64]. The bonds between V^4+^ and catechols are about half as strong as a covalent bond [70]. There is some experimental evidence for the usage of V^4+^ crosslinking in adding extra strength to the outer protective cuticle [70]. Thus, covalently crosslinked proteins are connected to one another through weaker metal–catechol coordination bonds. All these interactions contribute to the binding of byssal threads to solid surfaces.

### 5.2. Tunichrome-Based Biomaterials

**Figure 22:**
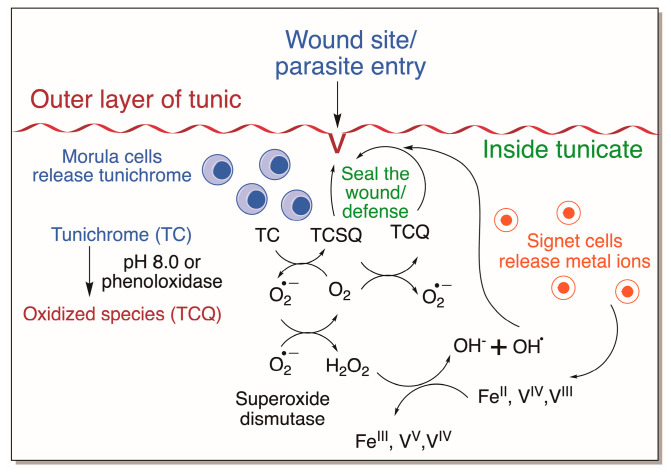
Tunichrome function in tunicates. Morula cells containing tunichrome (TC) released during injury or parasite invasion is oxidized by the phenoloxidase and/or exposure to pH 8 to the quinone form (TCQ). Interaction of tunichrome with oxygen produces tunichrome semiquinone radicals (TCSQs). Superoxide dismutase converts superoxide to oxygen and hydrogen peroxide. Interaction of hydrogen peroxide with reduced metals leads to hydroxyl radical production that is used for killing invading organisms. Oxidized species of tunichromes are used for wound healing and encapsulating the parasites. Not shown in figure—Tunichromes also bind to the outer tunic and allow its hardening.

For a long time, it used to be assumed that tunichromes and related compounds are used as metal chelators/transporters in tunicates. However, this popular belief was put to rest with the finding that metals and tunichromes are localized in different cell types of tunicates. Tunichromes and related compounds have been shown to possess antibiotic properties [25,26,28,29]. Their facile oxidation and easy production of reactive oxygen species account for part of the antibiotic properties. But as many investigators have noticed, they are extremely unstable and undergo facile oxidative polymerization. Lysis of the blood cells of tunicates leads to the production of a greenish black fluid, called Heinze precipitate, that frequently yields a dark and fibrous material [76,77]. Blood cells also contain phenoloxidase that readily oxidizes the tunichromes. Moreover, practically all tunichromes are pH-sensitive and rapidly undergo nonenzymatic aerial oxidation when exposed to the conditions of sea water, namely, pH 8. This and other observations lead to the proposal that tunichromes are not only involved in defense reactions, but also in wound healing and tunic formation [22,23,24,25,26]. Tunic, the outer shell of the tunicates, also contains abundant amounts of cellulose [78], which is not common to animals. Oxidized tunichromes could couple with each other and proteins, as well as the cellulosic fiber, forming hardened tunic that will protect tunicates [26]. Typically, tunichromes are localized in the morula cells and metal ions are accumulated in signet cells. During wounding, when these cells are exposed and ruptured, they release their contents. The presence of phenoloxidase as well as exposure to mildly alkaline pH initiates rapid oxidation of tunichromes and free radical production (Figure 22). This results in rapid deposition of Heinze precipitate and sealing of the wound. The same mechanism, in a controlled fashion, could be used for tunic formation as well (Figure 22).

### 5.3. N-Acyldopamine Derivatives and Cuticular Hardening

**Figure 23:**
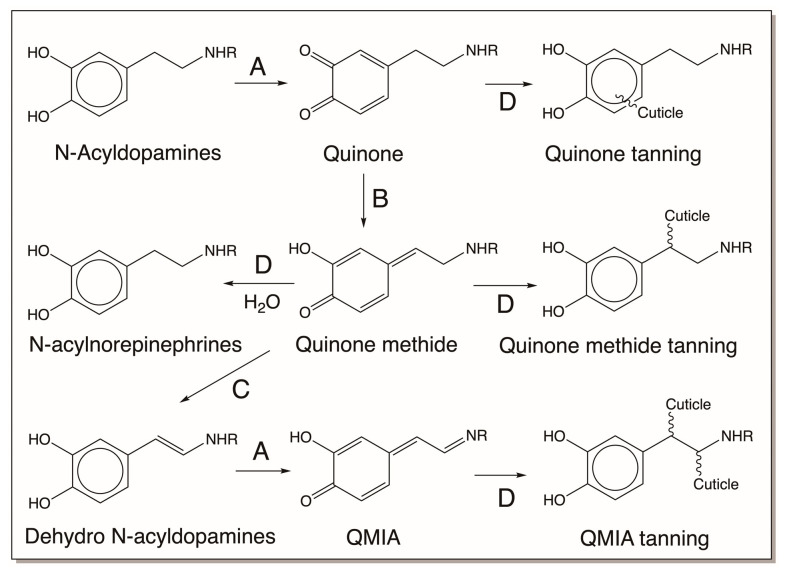
Unified mechanism for sclerotization of insect cuticle. N-acyldopamines (NADA, R = COCH_3_; NBAD, R = COCH_2_CH_2_NH_2_) are oxidized by cuticular phenoloxidases (A) to their corresponding quinones. Quinone isomerase (B) converts quinone to quinone methides, and the next enzyme, quinone methide isomerase (C), generates the side-chain desaturated N-acyldopamines. Dehydro-N-acyldopamines thus formed are further oxidized by phenoloxidases. Quinones, quinone methides, and QMIA are used for crosslink purposes, generating different adducts and crosslinks necessary to harden the cuticle. Part of the quinone methide also reacts with water to form N-acylnorepinephrines. (D = nonenzymatic reaction. Not shown in the figure are free-radical coupling reactions mediated by peroxidase and related enzymes, benzodioxan dimerization and oligomerization reactions, and metal chelating reactions that are possible for various catecholamines and their derivatives.)

Finally, we examine the biomaterial produced by the oxidation products of N-acyldopamines. The hard and often tanned exoskeleton is a vital body part of all insects which protects the soft-bodied animals from dehydration and harsh environmental conditions and invading parasites. A hard cuticle naturally does not allow for continuous growth. Therefore, insects and other arthropods often shed their old cuticle and make a fresh, larger one to accommodate the increased body mass. Freshly made cuticle is often very soft and pale. But in a matter of couple of hours, often, it becomes hard and sometimes darkened by what is known as sclerotization reactions. During sclerotization, catecholamine derivatives, mostly N-acyldopamines, NADA, and NBAD, are oxidized by cuticular phenoloxidases (both *o*-diphenoloxidase and *p*-diphenoloxidase, known as laccase) to reactive intermediates which are used to form adducts and crosslinking with cuticular proteins and chitin polymers. The protein–protein, protein–chitin, and chitin–chitin crosslinks thus formed provide the necessary hardening of the cuticle. Extensive studies carried out on the sclerotization of sarcophagid cuticle resulted in a unified mechanism for sclerotization of all insects, which is depicted in Figure 23 [16]. Not all mechanisms are shown in this figure. Both free-radical polymerization and metal chelation reactions also occur along with these reactions in different cuticles.

Different insect cuticles come with different tanned properties. The elastic cuticles of insects that can allow expansion and contraction in some insects are due to a crosslinked protein called resilin. The elastic protein resilin is crosslinked with dityrosine and trityrosine crosslinks that allow a layer of protein fibers to move one over the other during stretching and come back to their original size during contraction [12,13,14,15,16,79,80]. Some cuticles are very transparent and practically colorless, like dragon fly wings. They are also hardened by sclerotization reaction. Some cuticles or spots on cuticles that are jet black attain their color by eumelanin deposition [7]. Most of the cuticles are brown in color. Typically, brown-colored cuticles use NBAD as the major sclerotizing precursor, and colorless or lightly colored cuticles tend to use NADA as the sclerotizing precursor [16]. The hardness of the cuticle is attributed to the sclerotization reactions. Some sclerotized cuticles are very fragile, while some others can withstand heavy weight loads with ease. Not all the factors causing these differences are identified and more work is required to understand the details responsible for the wide range of biomechanical properties that various insect cuticles exhibit. Apart from cuticles, the mandibles of insects, which allow the crushing, cutting, and chewing of food materials, also seem to be hardened. This hardening is associated with the deposition metal ions, such as zinc [81]. Metal ions such as zinc accumulate in the hardened mandibles and jaws of invertebrates [75,81,82,83,84]. Thus, adding metal ions to organic composites modifies the hardening and toughness of biomaterials. In this regard, it is important to note that catecholamine crosslinks alone could provide very tough biomaterials. The stiff beak of the squid, *Dosidicus gigas*, which is made up of only organic materials (proteins and chitin, to be specific), contains many types of histidine catechol adducts that seem to confer stiffness and strength to the beak [82,83,84]. Finally, a variety of silks, egg cases, egg sacs, chorions, and oothecae of insects are also hardened and protected by various sclerotization and hardening processes [16,19]. Some are crosslinked by dityrosine-type crosslinks, and others are crosslinked solely by catecholamines and proteins and are devoid of chitin [19]. But the majority of hardening mechanisms in these novel biomaterials are yet to be unraveled.

Although melanin is often considered as a polymer of oxidation products derived from dopa and dopamine, its interaction with other biomolecules cannot be ignored, especially when dealing with insects and other arthropods [1,2,3,4,5,6,7]. Melanin in mammals is typically generated as exoskeletal pigmentation by melanocytes. On the other hand, insects and other arthropods generate melanin not only for exoskeletal pigmentation, but also for defense reactions and wound healing reactions practically in all body parts [16,85,86,87]. With its open circulatory system, producing melanin and its reactive precursors in the blood is highly dangerous and various control mechanisms play crucial roles in preventing and/or controlling damages caused by melanin-related compounds. But from a biomaterials point of view, the production of melanin–protein adducts, melanin–chitin adducts, and melanin–protein–chitin crosslinks is certainly a reality.

### 5.4. Synthetic Biomaterial Mimics

The ubiquitous use of catechols as crosslinking agents in nature has inspired volumes of work aiming to develop catechol-based polymers and hydrogels for a large variety of purposes, such as antifouling coatings, antibacterial coatings, catalysis, reduction of organic pollutants, oxygen reduction, lithium-ion batteries, solar cells, and supercapacitors, as highlighted in a recent review article [88]. In many of the applications, co-polymers are crosslinked through dopa or pyrogallol pendants and metal ions are added for reinforcement, mimicking, to some extent, the cohesion forces in the mussel foot. Oh et al. reported on a pyrogallol-conjugated chitin nanofiber composite and demonstrated a high shear strength under wet conditions [89]. Montoni et al. prepared a dopa-conjugated chitin-based polymer reinforced with Fe^3+^ crosslinking [90]. Kim et al. prepared a poly (dopamine acrylamide-co-n-butyl acrylate) crosslinked via phenyl diboronic acid [91]. This polymer was shown to be non-swellable and possess good self-healing properties in seawater.

The successful example of adhesion in a wet, dynamic environment provided by the mussel has inspired much research in developing an effective, nontoxic glue that could potentially be used in medical applications, such as wound closures and dental procedures [41,92,93,94]. Many researchers have explored the use of dopa-derived polymers for a variety of medical uses. Dopamine-linked primers consisting of methyl acrylamide and allyl- and thiol-containing compounds, as well as phenol–polyamine superglue, have been shown to be suitable for bone adhesion and tissue repair [90,91,92,93,94,95,96,97,98]. Mussel-mimetic tissue adhesives consisting of catechol derivatives linked to various other biomaterials have been demonstrated for internal medical uses, such as wound healing, bone fracture, and dental fixture [41,90,91,92,93,94,95,96,97,98]. The bulk of the work reported in the literature so far has focused on using the polymerization of dopa and/or dopamine-based materials. However, the resulting polymers tend to underperform in terms of mechanical strength [41,95] and adhesion in wet environments [98]. It has proven to be particularly challenging to control the deposition process and environment in a manner that is analogous to the carefully orchestrated process described above in terms of pH, the reducing environment, and the viscosity of the starting material [36,37,38,39]. Also, missing from most of these attempts is the diversity of chemistries that contribute to the adhesion of the mussel proteins to the surface.

Other approaches have focused on synthesizing several of the known byssal proteins to better emulate the natural system. One can use standard peptide synthetic strategies for this, but synthesizing proteins in the amounts necessary for industrial scale-up would not be cost-effective. Using *E. coli* to overexpress large quantities of the proteins is one option. However, *E. coli* does not have the machinery to carry out the necessary post-translational modifications of tyrosinase to dopa, and even when the enzymes that could perform this work are added to the reaction mixture, successful incorporation only occurs in less than 20% of the tyrosine residues in the protein. So, the strategy has shifted to the incorporation of non-natural amino acid substituents during translation. This can be achieved either through selective pressure incorporation [99,100] or by establishing an orthogonal translation system [101,102]. With selective pressure incorporation, the essential amino acid is depleted from the culture media, so that the desired derivative version (i.e., dopa in place of tyrosine) is selected in its place. With the orthogonal translation system, the genetic code is rewritten so that stop codons can be used to place noncanonical amino acids onto the growing protein. Enzymes that activate the insertion of amino acids are engineered to work effectively with the target noncanonical amino acid, such as dopa. Computation methods have been used effectively to find mutants that can insert the dopa units with impressive efficiency [102]. A high-throughput screening method has also been used to identify a suitable catecholamine polyphenol mix for nanocoating [103].

## 6. Summary and Conclusions

**Figure 24:**
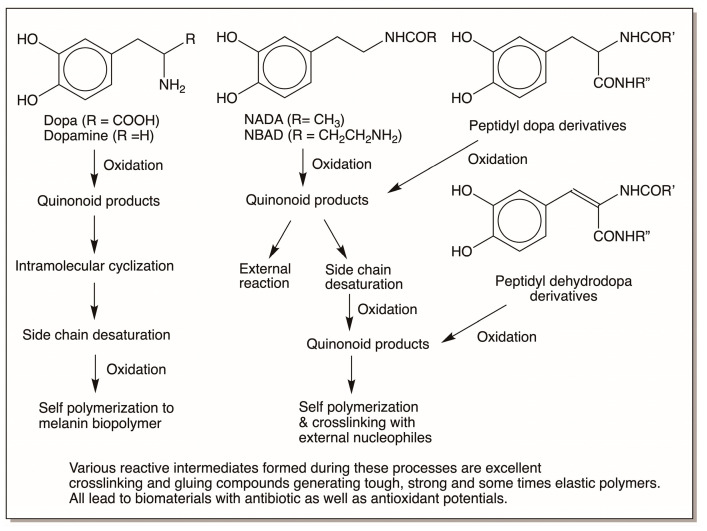
A bird’s-eye view of the reactions summarized in this review. Simple dopa and dopamine after the initial oxidation to their corresponding quinones often exhibit intramolecular cyclization and further oxidation to dihydroxyindolic compounds which suffer oxidative polymerization to produce melanin pigments. Although the reactive species formed in the pathway can exhibit external reactivities to a certain extent, mostly internal reactions seem to dominate in the case of dopa and dopamine. On the other hand, NADA and NBAD, which are major sclerotizing precursors for hardening and strengthening the exoskeletons of insects, mostly exhibit external reactivities and glue to structural proteins and carbohydrate polymers, generating different kinds of biomaterials. Peptidyl dopa derivatives as well as peptidyl dehydrodopa derivatives also show mostly external reactivities and produce biomaterials exhibiting different strengths and elasticities. All biopolymers formed from catecholamines are capable of exhibiting antioxidant as well as antibiotic properties to varying degrees.

Figure 24 offers a bird’s-eye view of the reactions outlined in this review. Dopa and dopamine upon oxidation generate their corresponding quinones. These quinones, due to the presence of suitably situated internal amino nucleophilic groups, exhibit mostly intramolecular cyclization reactions. External reaction is minimal and is only observed with thiols which lead to yellow to red pheomelanin polymers. The intramolecular cyclized products eventually undergo side-chain desaturation to form 5,6-dihydroxyindoles, which are highly unstable and exhibit rapid oxidative self-polymerization to produce eumelanin pigment that is essential to protect animals from damaging solar radiation. When the amino group is protected by acylation so that the internal reactions are prevented, the resultant catecholamines exhibit mostly external reactivities suitable for bonding and crosslinking structural proteins and carbohydrate polymers, producing exoskeletons with varying degrees of toughness, flexibility, elasticity, and strength. This is particularly useful to build the exoskeletons of arthropods. Similarly, dopa embedded in protein and peptide chains in the primary structure by post-translational modification of its tyrosine residues exhibits external reactivities after oxidation to its quinonoid product. In all these cases, the side chain of catecholamine can also exhibit desaturation either with the help of suitable enzymes or by spontaneous nonenzymatic reactions. Dehydrodopa and dehydrodopamine derivatives thus formed are far more reactive than their saturated counterparts and suffer rapid nonenzymatic oxidation to produce novel QMIA derivatives that are uniquely capable of directly crosslinking to nucleophiles and bonding them together. Furthermore, all catechols and their oxidation products are also suitable for chelating various metal ions found in the environment. These reactions provide additional strategies for strengthening various biomaterials. Finally, numerous catecholamine derivatives and their biopolymers can generate, as well as interact with, free radicals, providing both protective and damaging effects to the surroundings.

Thus, the remarkable and wide range of reactivities exhibited by catecholamine derivatives contribute significantly to the key functions of these biomolecules. The potentials of catecholamine-derived biomaterials to function as novel building blocks and adhesive molecules with antioxidant and antibiotic properties are also highly evident. The mechanisms by which these biomaterials function in a myriad of complex ways that are still being examined in several laboratories. So far, most laboratories have focused on only dopa/dopamine and a few other catechol-based polymers to develop novel biomaterials. Other small molecules as well as macromolecules based on the scheme of reactions outlined in this review will be useful for the production of newer and more accessible glues and polymers for future use in medical and industrial settings.

## Figures and Tables

**Table 1:** Names and chemical names of tunichromes and related compounds.

Compound Name	Chemical Name
Tunichrome An-1	Topa-Dehydrotopa-Dehydrotopamine
Tunichrome An-2	Dopa-Dehydrotopa-Dehydrotopamine
Tunichrome An-3	Dopa-Dehydrotopa-Dehydrodopamine
Tunichrome Pm-1	Topa-Topa-Dehydrotopamine
Tunichrome Pm-2	Dopa-Topa-Dehydrotopamine
Tunichrome Pm-3	Topa-Topa-Dehydrodopamine
Tunichrome Mm-1	Gly-Dehydrodopa-Dehydrodopamine
Tunichrome Mm-2	Leu-Dehydrodopa-Dehydrodopamine
Tunichrome Sp-1	Dopa-Dopa-Gly-Pro-Dehydrodopamine
Plicatamide	Phe-Phe-His-Leu-His-Phe-His-Dehydrodopamine
Clionamide 1	6-BrTrp-Dehydrotopamine
Celenamide A	Leu-Dehydrotopa-6-BrTrp-Dehydrodopamine
Celenamide B	Val-Dehydrotopa-6-BrTrp-Dehydrodopamine
Celenamide C	Leu-Dehydrotopa-6-BrTrp-Dehydrotyramine
Celenamide D	Leu-Dehydrotopa-Dehydrotopa-Dehydrodopamine
Celenamide E	Dehydrotopa-6-BrTrp-Dehydrodopamine
Morulin Pm	Polycyclic compound with 6-BrTrp & dehydrodopamine
Purpurone	Polycyclic compound with dehydrodopamine
Lamillarins	Polycyclic compounds with dehydrodopamine
Ningalins A-D	Polycyclic compounds with dehydrodopamine
Storniamides A-D	Polycyclic compounds with dehydrodopamine

## Data Availability

Not applicable.

## Abbreviations

DHI—5,6-dihydroxyindole; DHICA—5,6-dihydroxyindole-2-carboxylic acid; NADA—N-acetyldopamine; NBAD—N-β-alanyldopamine; QMIA—quinone methide imine amide; Dehydro NADA—1,2-dehydro-N-acetyldopamine; TC—tunichrome; TCQ—tunichrome quinonoid species; TCSQ—tunichrome semiquinone radical.
